# Composition profiling of inhomogeneous SiGe nanostructures by Raman spectroscopy

**DOI:** 10.1186/1556-276X-7-633

**Published:** 2012-11-21

**Authors:** Andrea Picco, Emiliano Bonera, Fabio Pezzoli, Emanuele Grilli, Oliver G Schmidt, Fabio Isa, Stefano Cecchi, Mario Guzzi

**Affiliations:** 1 L-NESS, Dipartimento di Scienza dei Materiali, Università degli Studi di Milano-Bicocca, via Cozzi 53, Milan 20125, Italy; 2 Institute for Integrative Nanosciences, IFW Dresden, Helmholtzstr 20, Dresden, 01069, Germany; 3 L-NESS, Dipartimento di Fisica, Politecnico di Milano, Polo Regionale di Como, Via Anzani 42, Como, 22100, Italy

**Keywords:** Raman, SiGe, Nanotechnology, Composition, Structural characterization, Raman efficiency

## Abstract

In this work, we present an experimental procedure to measure the composition distribution within inhomogeneous SiGe nanostructures. The method is based on the Raman spectra of the nanostructures, quantitatively analyzed through the knowledge of the scattering efficiency of SiGe as a function of composition and excitation wavelength. The accuracy of the method and its limitations are evidenced through the analysis of a multilayer and of self-assembled islands.

## Background

SiGe nanostructures [1] such as quantum dots and islands are appealing for applications in photonics [2], microelectronics [3], thermoelectrics [4], and possibly quantum computation [5]. The fabrication of these nanostructures is often accompanied by composition inhomogeneities, as in the case of Stranski-Krastanov grown self-assembled islands [6]. The composition profile is a crucial parameter for several functional properties, such as bandgap and mobility. This has boosted the need of techniques to gather access to the exact SiGe concentration profiles. Ge distribution within the nanostructures can be measured by transmission electron microscopy (TEM) [7], atomic force microscopy (AFM)-based nanotomography [8,9], and X-ray diffraction [6,10]. Raman spectroscopy is a commonly used technique for the structural characterization of homogeneous SiGe [11-14], and in this work, we demonstrate how it is possible to extend its capabilities to the extraction of compositional profiles, with the advantage over the aforementioned techniques of being simultaneously fast and nondestructive.

The Raman spectrum of SiGe (Si_1−*x*_Ge_*x*_) depends on the relative alloy Ge content *x*. Three Raman peaks can be observed and related to Si-Si, Si-Ge, and Ge-Ge vibrations: their relative intensity and frequency are functions of *x*. The Raman spectrum Ф of an inhomogeneous structure contains information about the internal composition profile, i.e., Ф is a convolution between the composition profile and a set of Raman spectra, ϕ_*x*_, of bulk SiGe alloys with constant composition *x*. The Raman spectra set, ϕ_*x*_, represents the amplitude of scattering as a function of the wave number. The convolution is weighted by the spatial distribution of the excitation light in the nanostructures, the collection geometry, as well as by *S*_*x*_, the Raman efficiency of the alloy, which is also dependent on *x* for a given excitation wavelength λ_exc_. [15]. We want to show that the Raman spectrum can be used to reconstruct the spatial distribution of composition within an inhomogeneous sample, a process which was not possible before the recent publication of the values of *S*_*x*_ in the work of Picco et al. [15].

We will present a procedure for individuating the different compositions contributing to Ф and also for the reconstruction of a composition profile. First, the method will be discussed in detail while being applied to a SiGe multilayer. The choice of this sample was based on the fact that it can be characterized precisely with other experimental procedures and therefore can represent the best benchmark for the determination of the method’s validity. After a detailed discussion on the multilayer, the procedure will be also applied to SiGe self-assembled nanoislands.

## Methods

**Figure 1:**
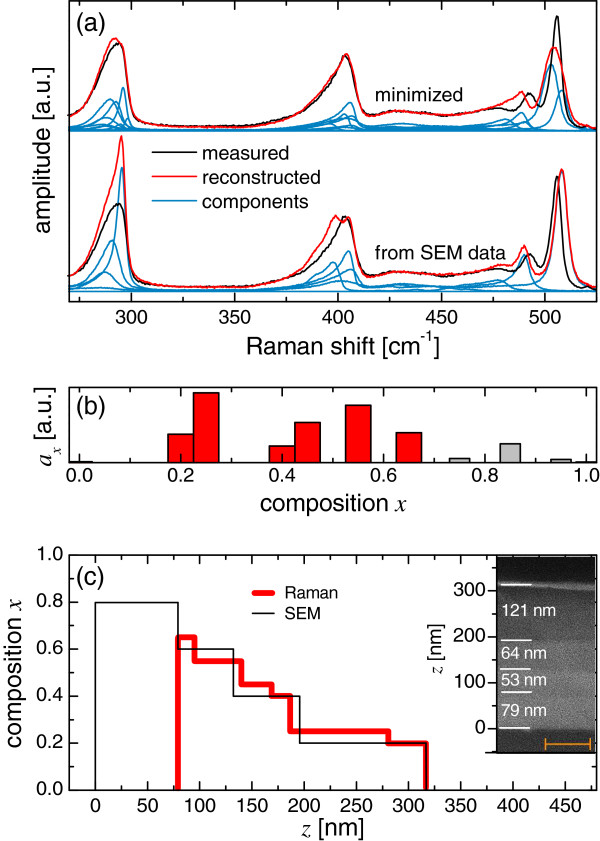
**Composition profiling of a SiGe multilayer.** (**a**) Experimental (black) and reconstructed (red) Raman spectra. The blue spectra are the weighted ϕ_*x*_ components. The upper reconstructed spectrum is the result of the minimization of Equation 1, while the lower reconstructed spectrum is generated from the thickness values obtained by SEM. (**b**) Relative spectral contributions, *a*_*x*_, with respect to the composition. (**c**) Composition profile of the multilayer given by the Raman measurement compared to the profile obtained by SEM. In the inset of (c) a cross-sectional SEM image (the scale bar is 100 nm) with the thickness of the layers is reported. The height from the substrate surface is *z*. The deepest layer, with *x* = 0.8, was not included in the Raman analysis (see text).

The multilayer sample consisted of a stack of four SiGe relaxed epitaxial layers deposited by low-energy plasma-enhanced chemical vapor deposition (LEPECVD) on Si(001) with composition *x* = 0.20, 0.40, 0.60, and 0.80 from the surface to the substrate (see the scanning electron microscopy (SEM) image in the inset of Figure 1c). The values of the composition measured by X-ray diffraction were 0.19, 0.40, 0.61, and 0.80.

**Figure 2:**
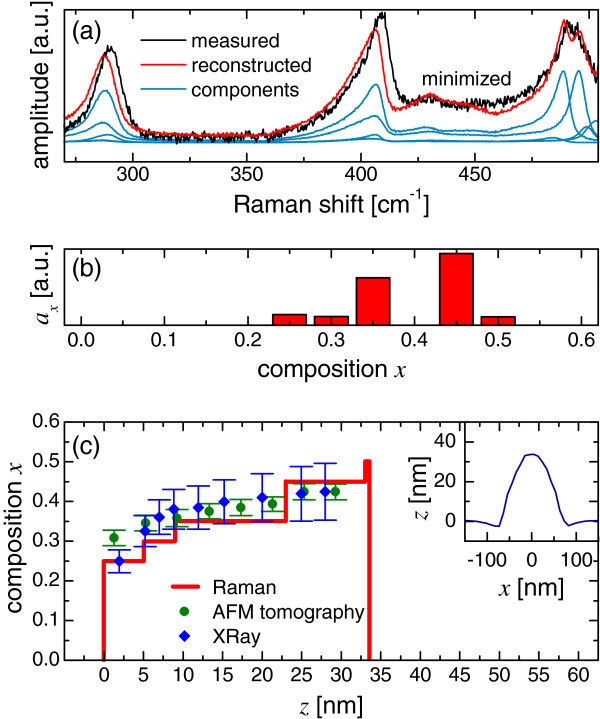
**Composition profiling of a SiGe island.** (**a**) Experimental (black) and reconstructed (red) Raman spectra. The blue spectra are the weighted ϕ_*x*_ components. (**b**) Relative spectral contributions, *a*_*x*_, with respect to the composition. (**c**) Composition profile inside each island modeled as a multilayer. The distance from the substrate surface is *z*. The uncertainty on the thickness values by Raman spectroscopy is about 10% of the total thickness. The data are compared to AFM and X-ray [18] results of similar islands. In the inset of panel (c), the AFM profile of one island is reported.

The sample of SiGe self-assembled islands was grown by molecular beam epitaxial deposition of 8.5 monolayers of Ge at 700°C. AFM measurements (inset of Figure 2c) on several islands show an ensemble of almost identical barn-shaped islands [16] with a very narrow height distribution of 13% full width at half maximum. The average diameter is 150 nm, and the average height is 35 nm. The islands are randomly arranged with a density of about 10 islands/μm^2^.

Raman spectra were acquired with a Jasco R800 double-additive spectrometer (Jasco Corporation, Tokyo, Japan) coupled to a microscope. All the spectra were acquired with an excitation wavelength, λ_exc_ = 532 nm. Notch filters were used to reduce the radiation at the same wavelength of λ_exc_*.* The resolution of the instrument was about 0.6 cm^−1^, a negligible value as compared to the natural bandwidth of all the Raman phonons at any composition. The spatial resolution achieved with an objective with a numerical aperture of 0.75 was about 1 μm. The incident power density was kept at less than 0.1 mW/μm^2^ in order to prevent the sample from heating (this value was determined by observing that lower powers did not modify the spectrum).

## Results and discussion

### Deconvolution of the Raman spectrum of the SiGe multilayer

We denote by the symbol Ф the experimental Raman spectrum of the multilayer, as shown in Figure 1a (black). Symbol Ф implicitly contains all the information about the composition profiling. Its deconvolution process starts by collecting an ensemble of a number *n* of ϕ_*x*_ spectra with 0 <*x* < 1. The collection must cover a spectral range *R*, including the three SiGe main Raman modes. Each ϕ_*x*_ must be normalized in order to have its integrated intensity on *R* equal to 1. The deconvolution of Ф is then obtained by finding the set of *n* coefficients *a*_*x*_ that minimize the quantity^a^ Δ_1_ defined as follows:

(1) $$\Delta_{1}={\left(\Phi -\sum a_{x}\phi_{x}\right)}^{2}\text{.}$$

The determination of the spectral contributions *a*_*x*_ consists in a deconvolution of the experimental spectrum, Ф, and represents an important added value with respect to the methods based on Raman spectroscopy found in previous works [12,17] where one must rely on the assumption to have a homogeneous sample. The set of *a*_*x*_ unfolds the presence of a distribution of compositions within the probed layer. In addition, the knowledge of *a*_*x*_ can be also the starting point for a reconstruction of the composition profile. We will demonstrate these features by analyzing a SiGe multilayer and SiGe self-assembled islands.

The deconvolution must be carried out starting from a set of ϕ_*x*_ that can be measured or taken from the work of Picco et al. [15]. The starting point of the procedure is a choice of a basis formed by a limited number of ϕ_*x*_ and therefore a relatively high value of Δ*x*. At this point, whatever the initial seeds, *a*_*x*_, the algorithm always converges to the same unique solution. The next step is to decrease Δ*x*, and therefore to increase the number of ϕ_*x*_, and repeat the algorithm. The process continues as long as shrinking Δ*x* leads to a decrease of Δ_1_, and the solution keeps on being unique. When the uniqueness condition is not satisfied, the solution of the algorithm is discarded, and the process ends. In the example reported here, we obtained the best spectrum with a unique solution using 21 ϕ_*x*_ spectra with a sampling interval Δ*x* of 0.05. The minimization of Equation 1 leads to the generation of a reconstructed spectrum Σ*a*_*x*_ϕ_*x*_ (Figure 1a, upper red spectrum) which most closely approximates Ф. The inspection of the *a*_*x*_ values shows that the spectrum has contributions from compositions around [0.20 to 0.25], [0.40 to 0.45], [0.55 to 0.65], and [0.75 to 0.95], as shown in Figure 1b. These *x* values are in agreement with the nominal values of composition within an accuracy Δ*x* ≈ 0.10, a figure that demonstrates the potential of this kind of analysis.

Notice that there are some limitations to the applicability of this procedure. This method cannot work in those nanostructures where a strong phonon confinement takes place, typically with dimensions of a few nanometers. In all other cases, the main problems related to the determination of *a*_*x*_ rise from mechanical strain. Since the ϕ_*x*_ spectra are acquired from unstrained bulk, the effect of strain on Ф cannot be taken into account from Equation 1. The small discrepancies in Figure 1a are indeed related to the small residual strain in the stack. Other works in the literature [12] show, for example, that a biaxial strain of the order of 1% modifies the spectral position of the Si-Si band like a change in *x* of about 10%. In this sample, we checked the residual strain by X-ray diffraction in the layers (+0.3%, +0.1%, +0.1%, and −0.1% from top to bottom); therefore, the success of the deconvolution of Figure 1 relies in the fact that the strain was small enough not to corrupt significantly the analysis. It is important to underline that Figure 1b was obtained from one single spectrum and shows clearly that Ф is a superposition of several contributions, shedding light on Ge inhomogeneous distributions in the sample: a result which is beyond the grasp of the optical methods reported in the literature.

### Composition profiling of the SiGe multilayer

The *a*_*x*_ values show that the spectrum is the result of a superposition of different spectra from different compositions, but they cannot be taken directly as a quantitative measure of the relative abundance of each composition. The reason is that the *a*_*x*_ values are influenced also by the Raman efficiency *S*_*x*_ and by the spatial attenuation of the excitation laser. Nevertheless, once *S*_*x*_ are known [15], it is possible to extract from the Ф decomposition further information about the inner structure of the sample, namely the thickness *d*_*x*_ of each layer displaying a constant composition. The test sample is a stack of layers, and we can look for the set of *d*_*x*_ that minimizes the quantity Δ_2_ defined as follows:

(2) $$\Delta_{2}=\sum {\left(\frac{a_{x}}{\sum a_{x}}-\frac{I_{x}}{\sum I_{x}}\right)}^{2}\text{,}$$

where *I*_*x*_ is a quantity proportional to the spectral contribution *a*_*x*_ and is expressed as follows:

(3) $$I_{x}=e^{-2m}\;S_{x}\;\left[L_{x}\left(1-e^{-2d_{x}/L_{x}}\right)\right]\text{,}$$

being *L*_*x*_ the laser penetration depth for the concentration *x*[17]. The quantity in square brackets is proportional to the integrated excitation of the single layer, where the excitation decreases as a function of depth *z* as exp(−*z*/*L*_*x*_), and factor 2 takes into account the self-absorption. Quantity *m* is defined as follows:

(4) $$m=\sum d_{x^{\prime }}/L_{x^{\prime }}\text{,}$$

where it must be summed up for all the layers *x′* above the layer *x* so that *e*^*−*2*m*^ represents the attenuation of the excitation and collection for the buried layer *x*. The minimization of Equation 2 leads to the determination of *d*_*x*_. In this part of the procedure, the main source of error is the knowledge of *L*_*x*_, especially for the buried layers, where this uncertainty is more relevant. This can be observed, for example, by plotting a spectrum reconstructed from the *d*_*x*_ values obtained by SEM (Figure 1a, lower red spectrum), while the Si-Si and Si-Ge modes are well reproduced because they are generated mostly by the upper layers; the simulation of the Ge-Ge mode is not accurate since it is generated by the lower buried layers. In addition, the condition *d*_*x*_ >>*L*_*x*_ must be false for all the layers involved in the simulation, i.e., each layer must be properly excited by the laser throughout all its thickness, and this represents another possible limitation of the technique. In this sample, the deepest layer did not satisfy this condition and was excluded from the minimization. The results are reported in Figure 1c (red line). During minimization, we fixed the total thickness of the stack *d*_stack_ and imposed that Ge-richer layers were below Si-richer layers. Within the region of the sample involved in the simulation, for each value of the vertical position *z*, the composition profile obtained with the Raman analysis matches the profile independently measured by SEM (Figure 1c, black) within the remarkable accuracy of 10%.

### SiGe nanoislands

In order to test its validity on different structures, we applied this method to the case of self-assembled SiGe islands grown by Stranski-Krastanov process on a flat Si(001) substrate. The experimental spectrum of the islands, as shown in Figure 2a, was analyzed with Equation 1. Each island is treated as a stack of SiGe homogeneous disks. The signal from the substrate is excluded from the algorithm. Contributions of compositions between 0.25 and 0.50 are detected, with two major components at 0.35 and 0.45, as shown in Figure 2b, indicating a quite homogeneous inner composition. In addition to the deconvolution, we tried to get a coarse indication of the vertical profile of *x* by approximating a SiGe island as a multilayer stack with high Ge content on top (therefore neglecting a lateral variation of composition). The composition profile was obtained through the minimization of Equation 2 with the only constraint on the total thickness given by the AFM profile. The results in Figure 2b show values of *x* which are compatible with those results that one expects are from islands grown at similar temperatures [18]. By comparison with the results in the literature [18], we observe that despite the roughness of the approximation within the islands, the vertical distribution of *x* is in agreement with the trend observed with other techniques. Furthermore, Figure 2c shows that in this experiment the effect of strain cannot introduce an artifact in the determination of the composition of more than 0.10. We underline that the profile was obtained with a nondestructive measurement from one single spectrum.

## Conclusions

In conclusion, we presented a procedure for the analysis of the Raman spectra of inhomogeneous SiGe samples. This procedure is not intended to replace other techniques such as X-ray or TEM, but rather, it intends to offer a fast and nondestructive alternative for the investigation of several different nanostructures with a diffraction-limited spatial resolution, with the possibility of investigating single isolated nanostructures. This procedure is able to detect the composition values present in the sample and can also give a composition profile of the structure. While other methods based on Raman spectroscopy require the assumption of a homogeneous structure and can measure simultaneously composition and strain, this method can measure composition inhomogeneity, but its main requirement is a low mechanical strain. We also analyzed the other possible errors and limitations due to confinement, opacity of the layers, and uncertainty in the knowledge of the optical functions. The validity and the limitations of the method were tested with the aid of a multilayer stack independently characterized with other techniques. The application of this procedure to the study of self-assembled SiGe islands yields results in agreement with those from other techniques available in the literature. The method can be effective in SiGe nanotechnology especially when it is necessary to face high yields of samples, and it can be applied to the structural characterization of any kind of nanostructures such as quantum wells, islands, nanowires, or nanocrystals.

## Endnote

^a^The minimization procedure can be carried out by commercially available routines, e.g., the implementation of the nonnegative least square problem minimization algorithm available in Matlab.

## Abbreviations

AFM: Atomic force microscopy; SEM: Scanning electron microscopy; TEM: Transmission electron microscopy.

## Competing interests

The authors declare that they have no competing interests.

## Authors’ contributions

AP, EB, EG, and MG carried out the Raman measurements and simulations and drafted the manuscript. FI and SC provided the SiGe stack together with the XRD analyses. FP and OGS provided the island sample with the AFM characterization. All authors read and approved the final manuscript.
